# Cost-Effectiveness of Health Risk Reduction After Lifestyle Education in the Small Workplace

**DOI:** 10.5888/pcd9.110169

**Published:** 2012-05-10

**Authors:** Jorie C. Allen, James B. Lewis, Anthony R. Tagliaferro

**Affiliations:** Author Affiliations: Jorie C. Allen, James B. Lewis, University of New Hampshire, Durham, New Hampshire.

## Abstract

**Introduction:**

Investigations suggest that worksite health promotions in large companies decrease employer health costs and the risk for chronic disease. However, evidence of the success of such programs in small organizations is lacking. The purpose of this study was to determine whether a worksite health promotion program improves health risk and is cost-effective for a small employer.

**Methods:**

Intervention (n = 29) and comparison (n = 31) participants from a 172-employee organization underwent health screening of risk factors for coronary heart disease at baseline (fall 2006) and at 12 months (fall 2007). The intervention group attended lifestyle education videoconferences and reported physical activity. We used the Framingham Risk Score to calculate risk of coronary heart disease. To calculate cost-effectiveness, we used direct employer costs of the program divided by either the relative reduction in low-density lipoprotein cholesterol or the absolute change in coronary heart disease risk.

**Results:**

At 12 months, low-density lipoprotein cholesterol, total cholesterol, and number of metabolic syndrome markers were significantly higher in the comparison group than in the intervention group. Total cholesterol was significantly lower at 12 months than at baseline in the intervention group. Waist circumference and number of metabolic syndrome markers increased significantly from baseline in the comparison group. Cost-effectiveness of the intervention was $10.17 per percentage-point reduction of low-density lipoprotein cholesterol and $454.23 per point reduction in coronary heart disease risk.

**Conclusion:**

This study demonstrated the cost-effectiveness in a small organization of a worksite health promotion that improved low-density lipoproteins and coronary heart disease risk in participating employees.

## Introduction

Five of the 10 leading chronic health disorders in the United States are linked to modern lifestyle factors (1). If risk factors for these disorders are identified, prevention is possible through lifestyle modification. An elevated low-density lipoprotein cholesterol (LDL-C) is associated with coronary heart disease (CHD) and frequently coexists with metabolic syndrome. LDL-C is the principal target for CHD risk reduction (2). Studies indicate that CHD risk is reduced at least 1 percentage point for every 1 percentage-point reduction in LDL-C (3-5).

Worksites are uniquely positioned to provide health promotion that successfully reduces risk for the development of chronic disease in employees (6-8). Employers are concerned about the financial impact of chronic disease, including their outlay for contributions to medical plan coverage, direct medical costs, absenteeism and sick leave, disability, worker’s compensation, and presenteeism costs (9).

Employees with modifiable lifestyle risk factors cost employers 228% more in direct medical costs than employees with lower risks (10). Moreover, the indirect costs related to absenteeism and presenteeism are also large, accounting for a large percentage of total health and medical care costs to employers. Many methods have been used to estimate the magnitude of such indirect costs, and the results of such analyses have varied. A review of the literature noted that the percentage of total cost (medical, pharmacy, absenteeism, and presenteeism) accounted for by presenteeism alone has been estimated to range from 14% to 73%, depending on the medical problem. For clinical disorders (type 2 diabetes, heart/circulatory disease, hypercholesterolemia, and obesity) amenable to lifestyle changes, presenteeism accounts for an estimated 28% to 56% of total costs (11). The relative importance of such indirect costs is likely to increase in the future, particularly for smaller employers that are less likely than larger employers to provide health coverage for employees and, therefore, are more likely to be affected by the indirect costs (12). Research suggests, however, that costs decrease when employee health risks are reduced through worksite health promotions (WHPs) (13). A meta-analysis on the cost savings of WHPs showed that the average annual employer cost of $144 per employee represented a cost savings of $358 per participant in 2009 dollars (14). Research on the prevalence of metabolic syndrome and its effect on health and worker productivity concluded that metabolic syndrome was associated with poor perceived health, higher absenteeism, and an increased trend of short-term disability, and noted that a WHP could be useful in reducing risk and lost productivity (15). Most of the studies that were evaluated involved organizations of more than 1,000 employees. Whether these findings can be generalized to smaller organizations is not known.

Most US employers are small organizations with fewer than 500 employees (16). The Small Business Administration reports that 80% of small businesses have 10 or fewer employees (17). Although small organizations make up the majority of US workplaces, a minority offers their employees a WHP. Many are unable to offer a WHP because of limited financial resources, and they lack the infrastructure of larger organizations (16). Hence, designing and testing the effectiveness of a WHP that is affordable to small employers are needed.

Research suggests that implementation of WHPs in small organizations presents challenges and opportunities. Small organizations often do not have staff expertise to support the development of a WHP, and because of limited resources, they tend to focus on programs mandated by regulatory bodies (18). In addition, small organizations typically operate with smaller profit margins, making it more difficult to support WHPs (19). On the other hand, small organizations may be able to implement WHPs more quickly than larger ones because of the shorter lines of communication and a stronger sense of community (19). Smaller organizations may be more efficient in the integration of wellness into their culture, achieving a measurable effect on a large portion of the workforce (20). The purpose of our study was to investigate whether a WHP in a small organization improved health risk factors of employees and was cost-effective to the employer.

## Methods

This intervention study compared the health risk factors of employees who received health risk screening plus lifestyle education (intervention) with those of employees who received screening plus minimal information (comparison). We collected data in September and October of 2006 and 2007 at 10 regional worksites of the same organization throughout the state of New Hampshire. The University of New Hampshire (UNH) institutional review board approved the study.

### Participant recruitment

Following recruitment of all employees of the University of New Hampshire Cooperative Extension (UNHCE) without exclusion, 64 of the 172 employees (37%, 6 men, 58 women, mean age, 48.9 y) from 10 worksites volunteered. UNHCE is a decentralized organization that has worksites in each of the10 counties in New Hampshire and on the UNH campus. Allocation of participants to each group depended on worksite; participants from 5 sites without access to videoconferencing were assigned by default to the comparison group. We randomized the remaining participants proportionally by site to either the intervention or comparison group, resulting in groups of 32. Participants signed an informed consent document and received no compensation. Before baseline screening, 4 participants withdrew from the study, leaving 31 comparison (4 men) and 29 intervention (2 men) participants at baseline. At 12 months, 29 comparison (4 men) and 26 intervention (2 men) participants remained (94% and 90% of baseline participants, respectively).

### Intervention

The intervention consisted of a lifestyle education program and the distribution of pedometers. The comparison group did not participate in either component. Ten monthly lifestyle education sessions were delivered using the Granite State Distance Learning Network (GSDLN). GSDLN is a partnership of organizations providing an interactive videoconferencing network in New Hampshire (http://gsdln.org/index.html). UNHCE offices that subscribe to the network have a meeting room equipped with the technology needed to conduct an interactive conference. Each session focused on a different health topic, such as CHD risk, diabetes, or hypertension, and emphasized the importance of a healthy diet and physical activity. An interactive question-and-answer session and sampling of foods prepared by volunteer facilitators followed the presentations. Foods chosen for their nutritional benefit and ease of preparation were whole-grain cereal products, meatless dishes, reduced-fat dairy products, healthy snack options, and various fruits and vegetables.

Intervention participants received new pedometers (Digi-Walker SW-401, Lees Summit, Missouri) and instructions to wear them during waking hours, to increase their steps to 10,000 or more daily, and to report their activity totals weekly. Studies suggest that 10,000 steps per day is a reasonable level for healthy adults to attain the health benefits of regular exercise (21). We examined data at each quarter, and we compared the mean number of steps for participants who reported steps for all 4 quarters with those who did not. (See Appendix for further details of the intervention.)

### Health screenings

All participants underwent health screenings, which took place after an overnight fast, at baseline and 12 months. We collected fingerstick blood samples and analyzed them with a portable analyzer (Cholestech LDX system, Cholestech Corporation, Hayward, California) for total cholesterol, high-density lipoprotein cholesterol (HDL-C), low-density lipoprotein cholesterol (LDL-C), triglycerides, glucose, and high-sensitivity C-reactive protein (CRP). We calculated the ratio of total cholesterol to HDL-cholesterol.

We used a battery-operated bioimpedance analyzer (BIA 450, Biodynamics Corporation, Seattle, Washington) to measure percentage body fat, percentage body water, and calculate body mass index. We measured height and weight using a Health-O-Meter 402 medical beam scale with stadiometer (Sunbeam Products, Inc, Bridgeview, Illinois), waist circumference using a flexible tape measure, and systolic and diastolic blood pressures (right arm, supine position) using a clinically approved automated monitor (Omron HEM-711 AC(N), Omron Healthcare, Inc, Bannockburn, Illinois).

Metabolic syndrome markers were defined by the standards of the National Cholesterol Education Program Adult Treatment Panel III (2). These markers included waist circumference greater than 40 inches for men and 35 inches for women, HDL-C less than 40 mg/dL in men and 50 mg/dL in women, triglycerides of at least 150 mg/dL, blood pressure of at least 135/85 mm Hg, and fasting blood glucose of at least 100 mg/dL. We calculated the 10-year risk for CHD by using the Framingham Risk Score (FRS), an established formula based on age, total cholesterol, HDL-C, systolic blood pressure, treatment of hypertension, and cigarette smoking (22). An FRS score of 1.4 equals a 1.4% risk of developing CHD in 10 years (22).

All participants received test results and discussed them with the lifestyle professional. All participants also received 7 health newsletters by e-mail. Each issue focused on a particular topic such as CHD, type 2 diabetes, or metabolic syndrome and was written in a style that was active, personal, and understandable (Appendix).

### Efficacy and cost-effectiveness

The primary measure of efficacy in this study was the relative percentage-point reduction in LDL-C. A secondary measure was the absolute change in CHD risk per FRS score. Computation of relative and absolute changes followed Soler et al (23):

Relative change = [(*I*post/*I*pre)/(*C*post/*C*pre) − 1] × 100%

Absolute change = (*I*post − *I*pre) − (*C*post − *C*pre)

where *I* = [mean value for intervention group], *C* = [mean value for comparison group], pre = baseline measurement, and post = 12-month measurement.

We calculated cost-effective ratios of the intervention from the perspective of the employer by dividing the cost of the intervention by either the relative percentage-point reduction in LDL-C or the absolute change in CHD risk (cost-effective ratio = cost of intervention/unit of effectiveness). Direct employer costs per capita were computed in 2006 dollars and included compensation for the intervention professional to present the educational material and administer the program, facilitation of remote site food demonstrations, teleconferencing services (including scheduling and technology support), and the cost of pedometers. We did not include the costs of developing materials. We also did not include costs for health screenings because they were equal for both groups.

### Statistical analysis

Statistical analyses used a general linear model with 1 exception; we examined differences in FRS by using Pearson χ^2^ analysis. Measures of high-sensitivity CRP were not normally distributed and, therefore, were log-transformed for parametric analysis. We used SYSTAT 12 (SYSTAT Software, Inc, Chicago, Illinois) for analyses. Statistical significance was set at *P* ≤ .05.

## Results

No differences in baseline measures were found between groups (Table). At 12 months, mean (standard deviation [SD]) LDL-C was significantly lower in the intervention group (110.9 [22.2] mg/dL) than the comparison group (126.7 [21.8] mg/dL), a relative difference of 13.4% between groups. LDL-C in the comparison group did not change from baseline to 12 months. Total cholesterol (mean [SD]) in the intervention group decreased significantly from baseline to 12 months, and at 12 months, it was 183.4 (22.2) mg/dL, significantly less than in the comparison group (198.6 [20.9] mg/dL). Total cholesterol in the comparison group was not different from baseline to 12 months. The waist circumference (mean [SD]) of the comparison group increased significantly from baseline (37.1 [2.2] to 38.9 [2.2] in), whereas in the intervention group, we found no change. No other within-group or between-group differences in health screening measures were found.

**Table:** Health Screening Test Results for Workplace Wellness Program Participants, University of New Hampshire Cooperative Extension, 2006–2007^a^

Variable	Comparison, Mean (SD)		Intervention, Mean (SD)	
	Baseline (n = 31)	12 months (n = 29)	Baseline (n = 29)	12 months (n = 26)
Age, y	48.5 (10.1)	48.5 (10.1)	51.7 (10.4)	53.7 (10.5)
Weight, lb	180.4 (48.6)	185.5 (48.7)	182.2 (52.8)	181.4 (51.5)
Body mass index, kg/m^2^	28.0 (7.6)	28.5 (7.7)	29.2 (7.9)	28.8 (8.1)
Waist circumference, in	37.1 (2.2)	38.9 (2.2)^b^	37.1 (2.3)	37.8 (2.4)
Body fat, %	31.1 (7.8)	30.3 (7.9)	31.7 (1.5)	31.2 (1.6)
Body water, %	49.5 (6.0)	50.4 (6.0)	49.3 (6.2)	49.4 (6.3)
Systolic blood pressure, mm Hg	125.6 (8.4)	131.0 (8.5)	125.6 (8.9)	130.9 (9.1)
Diastolic blood pressure, mm Hg	84.6 (11.5)	82.7 (11.5)	87.8 (11.8)	87.0 (12.1)
Total cholesterol, mg/dL	200.1 (20.8)	198.6 (20.9)	201.8 (21.7)	183.4 (22.2)^b^^,^ c
HDL-C, mg/dL	54.7 (16.9)	48.1 (17.0)	53.1 (17.6)	48.2 (18.0)
Triglycerides, mg/dL	145.7 (87.2)	153.8 (87.6)	156.7 (90.5)	142.8 (92.6)
LDL-C, mg/dL	121.0 (20.6)	126.7 (21.8)	122.3 (21.2)	110.9 (22.2)^c^
Total cholesterol/HDL-C	4.1 (1.3)	4.4 (1.4)	4.4 (1.4)	4.4 (1.4)
Fasting blood glucose, mg/dL	88.5 (7.1)	92.0 (7.1)	90.1 (7.6)	90.6 (7.7)
hsC-reactive protein, mg/L	2.2 (1.5)	2.8 (1.5)	2.2 (1.5)	2.9 (1.5)
No. of metabolic syndrome markers^d^	1.4 (0.6)	1.9 (0.5)^e^	1.5 (0.5)	1.3 (0.5)^f^
10-year risk for CHD^g^	1.4 (2.1)	1.8 (2.6)	1.8 (3.4)	1.9 (2.9)
Pedometer, steps per day	NA	NA	5,253 (1,644)	6,878 (1,645)^h^

Abbreviations: SD, standard deviation; HDL-C, high-density lipoprotein cholesterol; LDL-C, low-density lipoprotein cholesterol; hsC-reactive protein, highly sensitive C-reactive protein; CHD, coronary heart disease, NA, not applicable (comparison group did not receive pedometers).

a All variables except age, metabolic syndrome markers, and Framingham Risk Score corrected for age and sex. Statistical analyses performed by general linear model. Post hoc analysis performed by Tukey’s Honestly-Significant-Difference test.

b *P* = .001 within group.

c *P* = .01 between groups.

d Metabolic syndrome is a cluster of risk factors; for this study, we counted the following: large waist circumference, low HDL-C, high triglycerides, hypertension, and elevated fasting blood glucose.

e *P* = .02 within group.

f *P* = .002 between groups.

g Determined by Framingham Risk Score (23). Differences in scores examined using Pearson χ^2^ analysis.

h *P* = .05 within group.

The mean [SD] number of metabolic syndrome markers in the comparison group increased significantly from baseline to 1.9 [0.5] markers at 12 months, a value that was also significantly greater than that measured in the intervention group at 12 months (1.3 [0.5]). No within-group difference was found in the intervention group. The FRS at 12 months showed no significant between-group or within-group differences from baseline. However, the absolute reduction in CHD risk of the intervention group was 0.3 FRS points, a relative improvement of 18%.

Steps per day increased significantly from baseline (5,253 [1,644]) by the second quarter of the year (7,149 [1,648]) and continued through month 12 (6,878 [1,645]). Of 29 intervention participants, 20 reported steps at baseline. At 12 months, 13 of the remaining 26 intervention participants reported steps. We found no significant differences in the mean number of steps between participants who reported steps for all 4 quarters and those who did not.

Direct employer cost for the intervention was $136.27 per capita ($58.33 professional, $10.00 site facilitation, $35.00 videoconferencing, $32.94 pedometers). Cost-effectiveness of the intervention was $10.17 per percentage-point reduction in LDL-C and $454.23 per point reduction in CHD risk.

## Discussion

Findings of this study show that a small organization WHP can reduce the CHD risk factor LDL-C by 13% with cost-effectiveness that is greater than or comparable to that of large-scale WHPs. The reduction of LDL-C decreases the risk of heart disease (5). The calculated 1.4% CHD risk reduction per percentage-point reduction in LDL-C achieved in our investigation is comparable to that of other studies that identified the CHD risk-reduction benefit achieved per unit of LDL-C reduction (24). Furthermore, the number of metabolic syndrome markers of the intervention group stayed the same. These differences in risk factors occurred in a relatively healthy employee population.

Because the consequence of the failure to prevent disease is often treatment, we compare the economics of this study with those of pharmaceutical and lifestyle interventions. HMG-CoA reductase inhibitor drugs (statins) are agents for preventing CHD through LDL-C reduction. Although pharmaceutical and lifestyle interventions for hyperlipidemia are used for the primary prevention of CHD, lifestyle changes are the most cost-effective means to curb CHD risk (5). Evidence that lifestyle changes are more cost-effective than pharmaceutical treatment of LDL-C reduction is supported by the cost-effectiveness ratio of $10.17 per percentage-point reduction in LDL-C calculated in this investigation, which compares favorably with the $12 to $19 per percentage-point reduction in LDL-C reported for statin treatments (25).

The multicenter WISEWOMAN study (26) showed a cost-effective ratio of $470 per point reduction in the 10-year CHD risk and asserted that the program is a cost-effective approach for reducing CHD risk (27). Because WISEWOMAN examined the differences between a health screening group and a screening group plus educational intervention in CHD risk reduction, the $470 can be compared with our cost-effective ratio of $454.23. Therefore, we can claim that our intervention is cost-effective as well.

The primary component of this intervention was the simultaneous educational videoconferences in different worksites of a decentralized organization. Delivery of the lifestyle education accounted for most of the expense of the intervention. Potentially, this cost could be less per capita in future interventions, because additional videoconference attendees add no cost. Moreover, access to the videoconferences could be broadened. For example, educational sessions might be viewed on personal desktop computers at work; software allows for remote Internet viewing and archiving for later viewing. The use of technology can help to ease concerns about high costs and accessibility. Adaptation of WHP administration would be easier for small organizations, which have fewer layers of administration than larger organizations. Different types of workplaces and organizational structures may require flexible arrangements, such as delivery of lifestyle education during the typical lunch break, particularly if the organization is small and decentralized as in the present study.

The intervention group did not reach the stated goal of 10,000 steps per day. However, the 31% improvement over baseline corresponds to an average increase of nearly 1 mile per day in 50% or more of the intervention group. This improvement in daily steps appears consistent with the outcomes of other investigations (28).

This study has several limitations. First is the potential for self-selection bias among participants. Employees who were highly motivated to make lifestyle changes might have been more likely to volunteer than less motivated personnel. Also, the experimental design did not include a true randomized control group. Some of the comparison group participants had exposure in the workplace to intervention participants and, consequently, could have modified lifestyle behaviors. Because all participants received their test results and newsletters, undesirable results may have prompted private interventions outside of the research protocol, such as clinic visits and external health or fitness programs. It was beyond the scope of the study to control for outside influences that would modify behaviors. However, we were able to eliminate prescription medications as a confounder, because we examined data on medication use in both groups and found no differences between baseline and 12 months. Additionally, no new policies or amenities related to a healthy workplace environment are known to have been initiated during the time of the study. Another limitation was that our sample size was small; however, our participation rate was 35% of employees. Because a small organization limits the potential pool of participants, our study protocol did not exclude any volunteers according to the presence or absence of preexisting risk factors. Analysis of baseline characteristics, however, showed the population to be relatively healthy with no differences between groups in measures of CHD risk. The implications of our results are generalizable to a typical employee population.

Findings of our study demonstrate that LDL-C and overall CHD risk can be reduced through lifestyle education in a relatively healthy employee group in a small organization. A lifestyle intervention delivered at the worksite by videoconference is cost-effective, compared with statin administration or lifestyle education in a clinical setting. Such an intervention can also cost-effectively support change that could translate into overall health and cost benefits for both employers and employees of small organizations. If employers and employees are willing to participate in a WHP designed to improve the health of employees at reasonable cost, both parties will benefit.

## Appendix

### Appendix. University of New Hampshire Cooperative Extension Workplace Health Promotion Intervention

This document is available as a Microsoft Word file. [DOC - 451 KB]
