# Supplementary material for: Cost-Effectiveness of Health Risk Reduction After Lifestyle Education in the Small Workplace
Source: Prev Chronic Dis. 2012 May 10;9:E96. doi: 10.5888/pcd9.110169 (PMC3431951; doi:10.5888/pcd9.110169)
Supplement: Supplementary file 1 [file 11_0169_01.doc]

**Appendix**

**University of New Hampshire Cooperative Extension (UNHCE) Workplace Health Promotion (WHP) Intervention**

Before we allocated participants to experimental groups, we assessed each UNHCE worksite for membership in the Granite State Distance Learning Network (GSDLN, http://gsdln.org/index.html)*.* If GSDLN was not available, participants from those sites were assigned by default to the comparison group. Offices that did not have the videoconference network were Belknap, Carroll, Cheshire, Coös, and Hillsborough counties. Eighteen participants from these offices were allocated to the comparison group. The offices with videoconference access were Taylor Hall (UNH campus), Grafton, Hillsborough, Merrimack, Rockingham, and Sullivan counties. Because the number of volunteers from each of the offices that were part of GSDLN was different, the random selection of 14 comparison participants was done on a proportional basis, with at least 1 participant from each site assigned to the comparison group. This allocation resulted in comparison and intervention groups of 32 subjects each. After randomization but before baseline testing, 4 volunteers withdrew from the study, leaving at baseline 31 comparison and 29 intervention participants. At 12 months, 29 comparison and 26 intervention participants remained (94% and 90%, respectively) (Figure).

**Appendix Figure 1. Participant allocation and withdrawals, UNHCE Workplace Health Promotion, Fall 2006 – Fall 2007**


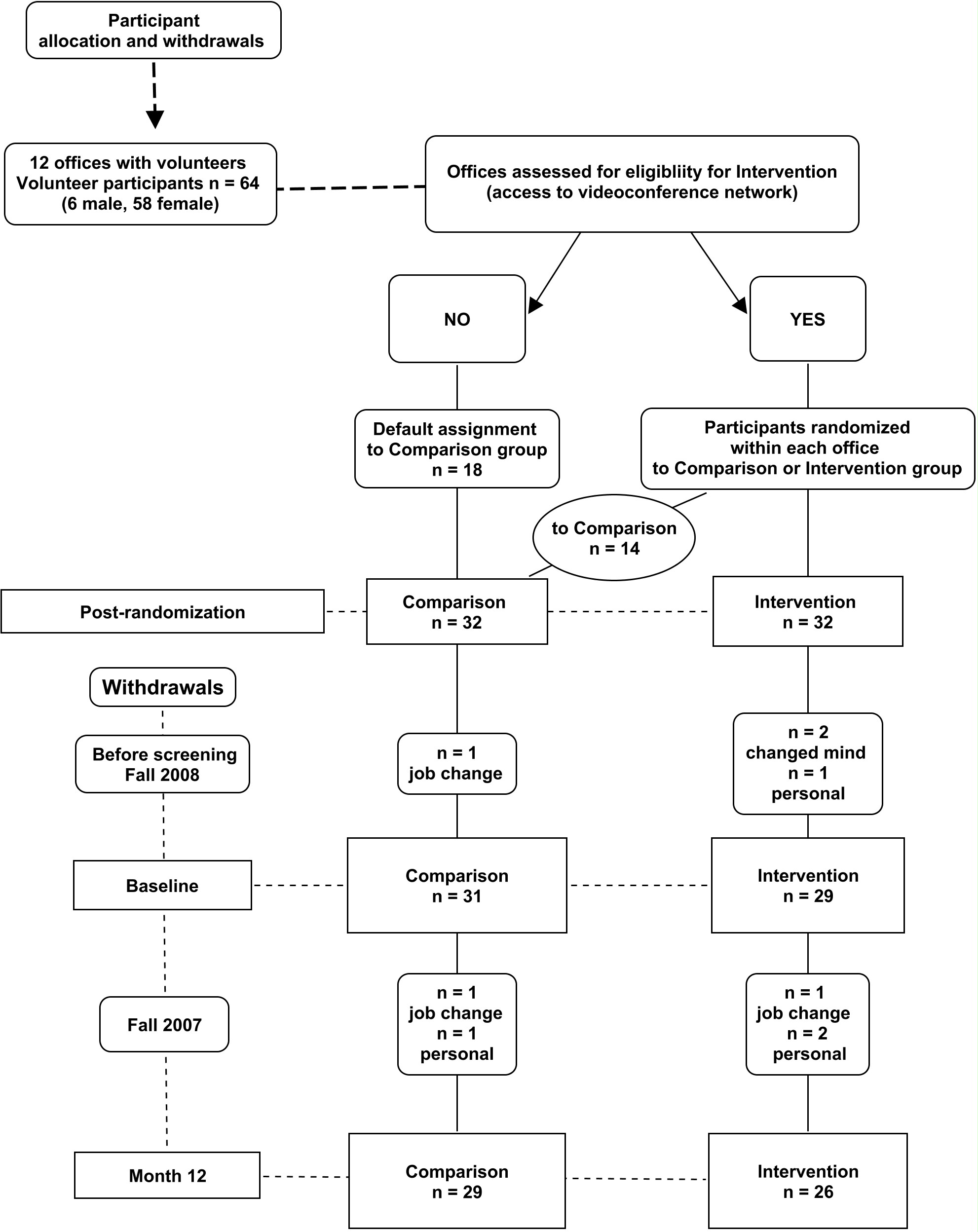


**Figure 1 Legend.** Before we allocated participants to experimental groups, we assessed each UNHCE worksite for membership in the Granite State Distance Learning Network (GSDLN, http://gsdln.org/index.html)*.* Eighteen participants from sites where GSDLN was not available were assigned by default to the comparison group. Because the number of volunteers from each of the offices that were part of GSDLN was different, the 14 comparison participants were randomly selected on a proportional basis, with at least 1 participant from each site assigned to the comparison group. This allocation resulted in comparison and intervention groups of 32 subjects each. After randomization but before baseline testing, 4 volunteers withdrew from the study, leaving at baseline 31 comparison and 29 intervention participants. At 12 months, 29 comparison and 26 intervention participants remained (94% and 90%, respectively).

Testing was conducted at county offices of UNHCE or on the UNH campus at baseline and at 12 months. All participants were tested for anthropometric, biochemical, and clinical variables. Participants were informed that the tests were not diagnostic; participants received and were able to discuss their results with a health professional (Registered Nurse and Health Coach). Discussion included the reason for the test in health risk identification, the target values, and the potential implications of undesirable results. Participants with values outside the target range were informed of the abnormality and then advised that further advice should come from their health provider. No counseling was provided. Please see Appendix Table 1 for a list of sources for target values for each test. All participants also received by e-mail 7 monthly newsletters that included links to resources related to health, wellness, and the particular topic focus of the newsletter (Table 2). Three examples of the newsletters are included in pdf format.

Members of the intervention group also were given pedometers, encouraged to walk 10,000 or more steps per day, and instructed to report weekly activity and step totals. A 2007 review of 26 studies of pedometer use among adults found that having either a goal of 10,000 steps per day or a personalized step goal was associated with a significant increase over baseline (1).

The lifestyle education was presented to intervention participants through GSDLN, which is a partnership of organizations providing an interactive videoconferencing network in New Hampshire. By using GSDLN, citizens can gain needed access to health education and community-based information not readily available in rural areas. Membership in GSDLN allows workers at separate worksites to meet, saving both time and expense, and is often used as a distance learning tool for employees (2). UNHCE offices that subscribe to the network have a meeting room equipped with the audio and video technology needed to conduct an interactive conference. The UNHCE criteria for selection as a GSDLN site included rurality and affordable access to broadband Internet connection with county support funds to sustain membership.

Each of the 10 monthly education sessions module was presented live, at lunchtime, in the form of a 30- to 35-minute PowerPoint presentation that focused on a different health topic.

Participants were asked to attend at least 7of the 10 educational sessions. This goal was achieved and each worksite was represented by at least 50% or more of participants at every session. The sessions emphasized the benefits of optimum nutrition and lifestyle on modifiable risk factors for chronic disease. The educational sessions were knowledge- and awareness-driven, because adult learners usually follow up new information with “why” questions. The concept that obesity is undesirable, for instance, would be followed up with the reasons why. Questions were encouraged and responses to questions were given by 1 of the authors (JA) who coordinated the WHP. Participants were encouraged to e-mail the program coordinator with additional questions and to offer feedback and suggestions. The following are examples of questions from participants:

- If cholesterol is bad, then why would you want to *increase* HDL-cholesterol?
- Should I take a (vitamin, mineral, omega-3) supplement?
- Do I have to work out for an hour a day to get benefit from physical activity?
- I’ve been told I have prediabetes. What is that and why don’t I need medicine?

The presentations were followed by food tastings provided by 1 volunteer participant at each site at the direction of the project manager. The purpose of the food tastings was to introduce the participants to healthy foods that might be unfamiliar or be perceived as unappetizing. Foods were chosen for their nutritional benefit and ease of preparation. Examples include whole-grain cereal products, meatless dishes, reduced-fat dairy products, healthy snack options, and a variety of fruits and vegetables (Appendix Table 3).

One of the specific challenges to the implementation of this study was the decentralization of the worksites, being located in 9 different counties in the state of New Hampshire. Even though each worksite is unique, all of them operate under the same organizational policies and guidelines. For example, vending machines are not present in the workplace, but some sites may be closer to an adjacent office with vending machines than others; elevators/stairs are present in some sites and not in others; some are located near sources of fast food, while others are not. All of the worksites had frequent meetings with snacks and beverages. No known initiatives for wellness, healthy eating, or physical activity are known to have been implemented during the time of the study. Outside of the UNHCE community, other groups have public wellness fairs where blood pressure or cholesterol is checked, and pedometers are distributed. However, there were no such events either sponsored by or on the premises of any of the worksites involved in the study. There are multiple exposures to positive and negative influences on health. It is not possible to control for the consumption of these influences among participants other than to have a comparison group with free access to the same environment.

Other practitioners or employers who are interested in developing a WHP have several options, including for-profit vendors of wellness programs. Such products may not be suitable for the smaller organization. Guidance on developing or enhancing a WHP can be found on several online sources. Included in these options are the Centers for Disease Control and Prevention (http://www.cdc.gov/workplacehealthpromotion), the American Heart Association (http://www.heart.org), the United States Workplace Wellness Alliance (http://www.uswwa.org/), and the Wellness Council of America (http://www.welcoa.org/).

The costs of screening and follow-up in this study were not included in the cost-effectiveness analysis. These tests were performed for research purposes; however, the WHP can be implemented in the absence of testing. All of the participants in this study had seen a health care provider in the last 5 years and 95% of the intervention group had seen a health care provider in the 12 months preceding baseline testing (data not shown). Testing for lipids/glucose, height, weight and blood pressure was carried out in these health checks. These costs are already borne by the employer and the employee in health-care premiums, deductibles, and co-pays. However, we recognize that some employers or practitioners might want to include such testing as part of a WHP. Costs of nondiagnostic screening tests performed by trained personnel are reasonable. For example, in 2006, the Center for Health Enhancement on the UNH campus offered the same evaluation as in our study at an insurance-reimbursable fee of $75.00.

**References**

1. Bravata DM, Smith-Spangler C, Sundaram V, Geinger Al, Lin N, Lewis R, et al. Using pedometers to increase physical activity and improve health. JAMA 2007;298(19):2296-304.

**Appendix Figure 1. UNHCE Workplace Health Promotion Participant Allocation and Withdrawals**

| **Appendix Table 1. Sources of Target and Health Risk Test Values UNHCE Workplace Health Promotion** | |
| --- | --- |
| **Measures** | **Source** |
| Weight  Body Mass Index | Clinical Guidelines on the Identification, Evaluation, and Treatment of Overweight and Obesity in Adults: The Evidence Report^1^ |
| Percentage Fat Mass | Guidelines from the American Council of Exercise^2^ |
| Waist Circumference  Blood Pressure  Fasting Blood Glucose  Total Cholesterol  LDL-Cholesterol  HDL-Cholesterol  Triglycerides  Metabolic Syndrome | National Cholesterol Education Program Expert Panel on Detection, Evaluation, and Treatment of High Blood Cholesterol in Adults (Adult Treatment Panel III)^3^ |
| Diet  Physical Activity | US Department of Agriculture and US Department of Health and Human Services 2005 Dietary Guidelines for Americans, Dietary Reference Intakes (DRI) and Recommended Dietary Allowances (RDA)^4^ |

^1^  Clinical Guidelines on the Identification, Evaluation, and Treatment of Overweight and Obesity in Adults: The Evidence Report. National Heart Lung and Blood Institute, National Institutes of Health Publication No. 98 – 4083. 1998.

^2^ Muth ND. American Council on Exercise (ACE) Guidelines for body fat percentage. American Council on Exercise. 2006. www.ace.org.

^3^ Executive Summary of the Third Report of The National Cholesterol Education Program (NCEP) Expert Panel on Detection, Evaluation, and Treatment of High Blood Cholesterol in Adults (Adult Treatment Panel III). JAMA 2001;285:2486-97.

^4^ Dietary Guidelines for Americans 2005. Committee for Dietary Guidelines for Americans, US Department of Health and Human Services, US Department of Agriculture, 2000.

| **Appendix Table 2. UNHCE Workplace Health Promotion Newsletters** | |
| --- | --- |
| **Volume (Issue)** | **Topics/ Contents**  (Online Resources and Exercise Calendar in each issue) |
| **1 (1)** | - What are the benefits of regular physical activity? - How to stay hydrated - If the shoe fits… - Your steps count - Tips for staying physically active - Take it slow |
| **1 (2)** | - Wellness: what’s in it for you? - Metabolic syndrome, heart disease, diabetes - Strategies for dealing with the holidays - Holiday recipe - Alcohol intake during the holidays |
| **1 (3)** | - Resolutions for a healthy year - Components of a healthy diet: Carbohydrates - Simple vs. complex carbohydrates - Whole grains and fiber - Recipe |
| **1 (4)** | - Dietary fats: why we need them - Good ones, not-so-good ones - Ways to add healthy fats to your diet - Recipe |
| **1 (5)** | - What are proteins? - Sources of protein - Meat and meat alternatives - Ways to add protein to your diet - The ‘skinny’ on high protein diets - Recipe |
| **1 (6)** | - What is osteoporosis? - What are the risk factors? - Dietary sources of calcium - Calcium supplements - Recipe |

| **Appendix Table 3. UNHCE Workplace Health Promotion Educational Sessions**  **Created and presented by Jorie Allen, RN, MS, WHP Coordinator** | |
| --- | --- |
| Introduction/ Proper footwear | - Introduction to Workplace Health Promotion - Schedule for sessions - Choosing the proper exercise shoe for you^1^ - Question-and-answer session |
| Physical Activity/Pedometers/ Hydration | - Benefits of regular physical activity - Keeping hydrated - Why use pedometers - Pedometer questions - Smart holiday choices - Question-and-answer session - Snack: green tea |
| Healthy Living/Healthy Eating/Healthy Body | - How lifestyle makes a difference - Get ready for the holidays - Obesity facts and statistics - Comorbidiies of obesity: Cardiovascular disease, type 2 diabetes, metabolic syndrome - Energy balance for weight loss - The holidays and your health: alcohol - Holidays at the office - Question-and-answer session - Snack: spiced oranges |
| Carbohydrates | - Diet resolutions - What is a healthy diet? - Carbohydrates - Simple vs complex - Whole grains/fiber - Low-carbohydrate diets - Question and answer session - Snack- whole grain crackers, breads with hummus |
| About Fats – some are essential, some are healthy, some are not | - What fats are and why we need them in our body. - The good ones and the not so good ones. - Benefits of healthy fats. - Easy ways to add healthy fats to your diet! - Question-and-answer session - Snack: low-fat or fat-free cheeses |
| Protein and Meat Alternatives | - What are proteins? - Sources of protein - Meat and meat alternatives - Importance of non-meat sources of proteins - Easy ways to add healthy protein to your diet - Question and answer session - Snack: meatless chili |
| Healthy Bones^2^ | - Implications of osteoporosis - Risk factors - Diagnosis - Current and future treatment - Resistance exercise - Question and answer session - Snack: Spinach dip with fat-free sour cream |
| Stress | - Prevalence of stress - The body’s reaction to stress - Risks of chronic stress - Connection between stress and sleep - Connection between stress and exercise - Coping with stress - Question-and-answer session - Herbal tea |
| Fruits, Vegetables, and “Functional” Foods | - Fruits and vegetables reduce disease risk - Antioxidants and phytochemicals - How much should I eat? - “What’s that?” quiz ( novel fruits and vegetables) - Tips to eat more (fruits and vegetables, that is!) - Question-and-answer session - Snack: star fruits, Asian pears, rainbow carrots |
| ^1^Presented by Certified Athletic Trainer  ^2^Presented by guest graduate student in Nutritional Sciences | |
